# WE CAN’T AVOID IT. IT’S THERE! AGEISM EXPERIENCED BY DIVERSE CULTURAL GROUPS IN OTTAWA, CANADA

**DOI:** 10.1093/geroni/igz038.924

**Published:** 2019-11-08

**Authors:** Caroline D Bergeron, Martine Lagacé

**Affiliations:** 1 Bexar County Community Health Collaborative, San Antonio, Texas, United States; 2 University of Ottawa, Ottawa, Ontario, Canada

## Abstract

Introduction: Discrimination based on age is pervasive across Canada. Little is known about the experiences of ageism among diverse cultural groups. The purpose of this pilot study was to explore the perceptions of ageism among culturally diverse older adults in Ottawa, Canada. Methods: Three focus groups were conducted with Chinese, Arab, and Indian older adults in Ottawa in June 2016. An 8-item protocol was developed to guide the discussions. Qualitative data were analyzed using open, axial, and selective coding. Results: Twenty-five culturally diverse older adults (9 Chinese, 6 Arab, and 10 Indian) participated in the focus groups. All described personal positive and negative examples of discrimination based on their age without being familiar with the term “ageism”. Several described their experiences with the intersection of age, race, and gender, although these interpretations varied by cultural group. Ageism in the media was also easily recognized. Participants recommended using specific content, communication channels, and organizations to counteract ageism. Discussion: This pilot study helped to illustrate that ageism is a societal problem that requires a societal solution. As Canada’s population becomes older and more diverse, important efforts are needed to raise awareness of ageism.

